# Dysregulation of the miR-557/MELK axis and its correlation with tumor markers in cervical cancer

**DOI:** 10.1093/labmed/lmag085

**Published:** 2026-10-26

**Authors:** Ning Liu, Sanqiang Niu, Xiuli Sun, Huaili Xin

**Affiliations:** Department of Obstetrics and Gynecology, Bozhou Hospital of Anhui Medical University, Bozhou, Anhui Province, China; Department of Obstetrics and Gynecology, Bozhou Hospital of Anhui Medical University, Bozhou, Anhui Province, China; Department of Obstetrics and Gynecology, Bozhou Hospital of Anhui Medical University, Bozhou, Anhui Province, China; Department of Obstetrics and Gynecology, Bozhou Hospital of Anhui Medical University, Bozhou, Anhui Province, China

**Keywords:** *miR-557*, *MELK*, SCCA, CA125, cervical cancer

## Abstract

**Introduction:**

Cervical cancer is a common gynecologic malignancy, and increasing evidence indicates that micro-RNA and its target genes are involved in its regulation. This study aimed to investigate alterations in *miR-557* and maternal embryonic leucine zipper kinase (*MELK*) expression and their associations with tumor markers in cervical cancer.

**Methods:**

Quantitative reverse transcriptase–polymerase chain reaction was used to quantify serum *miR-557* and *MELK* expression. Concurrently, a chemiluminescence immunoassay was performed to measure serum squamous cell carcinoma antigen (SCCA) and cancer antigen 125 (CA125) levels. Pearson correlation analysis was used to evaluate the associations among these biomarkers, and receiver operating characteristic curve analysis was performed to assess diagnostic performance. A dual-luciferase reporter assay was used to validate the targeting relationship between miR-557 and *MELK*.

**Results:**

Serum *miR-557* expression was statistically significantly lower in patients with cervical cancer, whereas *MELK* messenger RNA expression was significantly higher (*P* < .05). Pearson correlation analysis showed that *miR-557* expression was weakly negatively correlated with SCCA and CA125, whereas *MELK* messenger RNA expression was weakly positively correlated with SCCA and CA125 (*P* < .05). Our receiver operating characteristic curve analysis yielded a combined area under the curve of 0.909 for *miR-557* and *MELK* in the diagnosis of cervical cancer. The dual-luciferase reporter assay confirmed that *MELK* is a direct target of *miR-557*.

**Discussion:**

Reduced *miR-557* expression and elevated *MELK* expression were correlated with SCCA and CA125 levels in cervical cancer. By targeting *MELK*, *miR-557* may suppress cervical cancer progression, supporting the potential diagnostic and therapeutic relevance of the *miR-557/MELK* axis.

## Introduction

Cervical cancer is the second-most common malignant tumor of the female reproductive system.^1^ In 2020, an estimated 60 400 new cases and 34 200 deaths were reported globally, making cervical cancer the fourth-leading cause of cancer-related mortality among women.^2^ In China, mortality from cervical cancer has not yet declined to similarly favorable levels.^3^ Several factors contribute to the development of cervical cancer, including age, number of sexual partners, poor sexual hygiene, smoking, prolonged oral contraceptive use, nutritional status, obesity, genetic predisposition, and family history.^4^^,^^5^ However, infection with human papillomavirus (HPV) is a major risk factor for cervical cancer.^6^ Considerable research has been conducted attempting to elucidate the pathogenesis of cervical cancer. Serum tumor markers used in clinical practice, including squamous cell carcinoma antigen (SCCA) and cancer antigen 125 (CA125), together with other laboratory indicators, have contributed to the clinical management of cervical cancer.^7^^,^^8^ Although current screening approaches are beneficial for early detection, more sensitive and specific molecular biomarkers are still needed.

MicroRNAs (miRNAs) are a class of noncoding RNAs involved in the regulation of diverse cellular processes.^9^ Dysregulated miRNA expression has been associated with the development of various diseases, and accumulating evidence is indicating that aberrantly expressed miRNAs can contribute directly to tumor development. Previous studies have shown that *miR-557* expression is substantially decreased in cervical cancer tissues as well as in pancreatic cancer, hepatocellular carcinoma, and lung cancer, suggesting its potential value in the early diagnosis of cancer.^10‐12^ However, studies investigating *miR-557* in cervical cancer remain limited, and little is known about the potential value of serum *miR-557* as a prognostic marker. Therefore, this study aimed to investigate the role of *miR-557* in the initiation and progression of cervical cancer and to explore its potential molecular mechanism, with the goal of identifying potential molecular targets for the clinical diagnosis and treatment of this disease.

Maternal embryonic leucine zipper kinase (*MELK*) is a conserved, cell cycle–dependent serine/threonine protein kinase encoded on chromosome 9p13.2.^13^ It is highly expressed in various malignant tumors, including cervical cancer, ovarian cancer, hepatocellular carcinoma, breast cancer, acute leukemia, and myeloma.^14‐19^ However, the upstream regulation of *MELK* by *miR-557* in cervical cancer has not been well characterized. Clarifying this relationship may improve our understanding of the molecular mechanisms regulating *MELK* during the development and progression of cervical cancer and may provide a basis for more targeted therapeutic strategies.

In this study, the expression levels of *miR-557* and *MELK* were evaluated in clinical peripheral serum samples, and their associations with blood-based tumor markers of cervical cancer were analyzed. Statistical analyses and bioinformatics approaches were also used to evaluate *miR-557* and *MELK* expression and to examine the relationship between these 2 molecules. In addition, cell experiments were performed to assess *MELK* expression following *miR-557* overexpression or inhibition, further characterizing the regulatory relationship between *miR-557* and *MELK*. These findings may provide further insight into the molecular mechanisms underlying cervical cancer and identify potential targets for the disease’s diagnosis and treatment.

## Methods

### Patient information

This study included 35 patients with cervical cancer who had undergone surgical treatment at our hospital from December 2021 to August 2024 (cervical cancer group). In addition, 35 healthy women who had undergone physical examinations at our hospital during the same period were included as the control group. The diagnosis of cervical cancer was confirmed by histopathologic examination. None of the patients had received chemotherapy, radiotherapy, targeted therapy, or other cytotoxic drugs before blood sample collection. To ensure cohort homogeneity, patients with nonsquamous histologic subtypes of cervical cancer, including adenocarcinoma, adenosquamous carcinoma, and small cell neuroendocrine carcinoma, were excluded; thus, only patients with squamous cell carcinoma were enrolled. Individuals with clinically significant comorbidities, including severe hepatic or kidney dysfunction, coagulation disorders, acute infections, active systemic inflammation, severe anemia, or unstable cardiovascular conditions, as determined by routine preoperative laboratory testing and clinical evaluation, were also excluded to minimize potential confounding effects on serum biomarker measurements and ensure participant safety. Clinical data were obtained from the patients’ medical records. All participants provided written informed consent, and participation in the study did not impose any restrictions on treatment or cause any adverse effects.

### Sample collection and processing

Peripheral blood samples (5 mL) were collected from all participants in yellow-top vacuum blood collection tubes (Zhengzhou Taili Medical Devices Co, Ltd, Zhengzhou, China) in the morning after an overnight fast. The samples were subsequently centrifuged at approximately 1500 g for 5 minutes, and the resulting serum was collected for subsequent analyses. One milliliter of serum was transferred to a cryovial containing 2 mL pre-prepared RNA stabilizer (TIANGEN) and mixed manually, while another 1-mL aliquot was reserved for CA125 and SCCA measurements. All processing procedures were completed within 2 hours of sample collection. The samples were then stored at −80 °C until analysis.

### SCCA and CA125 measurements

Serum SCCA and CA125 levels were measured using a MAGLUMI X8 fully automated chemiluminescence immunoassay analyzer and the corresponding assay kits (Snibe Diagnostic). The measurements were performed using a chemiluminescence immunoassay. The cutoff values for positivity were SCCA at or above 2.5 ng/mL and CA125 at or above 35 U/mL, based on the manufacturer’s recommendations and the standard clinical practice of the Department of Laboratory Medicine at our hospital.

### RNA isolation and quantitative reverse transcriptase–polymerase chain reaction

Total RNA was isolated from peripheral serum using an RNA Isolater Total RNA Extraction Kit (Vazyme), and RNA purity was assessed. For miRNA expression analysis, reverse transcriptase was performed using an miRNA First-Strand cDNA Synthesis Kit (Vazyme) with specific stem-loop primers. Quantitative reverse transcriptase–polymerase chain reaction (RT-PCR) was subsequently performed using miRNA Universal SYBR qPCR Master Mix (Vazyme) on a light instrument under the following cycling conditions: 95 °C for 5 minutes, followed by 40 cycles of 95 °C for 10 seconds and 60 °C for 60 seconds. *U6* was used as the reference gene for miRNA expression. For *MELK* messenger RNA (mRNA) expression analysis, reverse transcriptase was performed using HiScript III All-in-One RT SuperMix Perfect for qPCR (Vazyme), followed by quantitative RT-PCR using Taq Pro Universal SYBR qPCR Master Mix (Vazyme). The amplification conditions were 95 °C for 5 minutes, followed by 40 cycles of 95 °C for 10 seconds and 60 °C for 30 seconds. Glyceraldehyde-3-phosphate dehydrogenase (GAPDH) was used as the internal control. Relative RNA expression levels were calculated using the 2^-ΔΔCt^ method. The specific primer sequences are listed in Table 1.

**Table 1:** Primer sequences.

Gene	Primer	Sequence (5ʹ-3ʹ)
*miR-557*	RT primer	GTCGTATCCAGTGCAGGGTCCGAGGTATTCGCA CTGGATACGACAGACAA
	Forward primer	GTTTGCACGGGGGGCC
	Reverse primer	AGTGCAGGGTCCGAGGTATT
*U6*	Forward primer	CTCGCTTCGGCAGCACA
	Reverse primer	AACGCTTCACGAATTTGCGT
*MELK*	Forward primer	GCTGCAAGGTATAATTGATGGA
	Reverse primer	CAGTAACATAATGACAGATGGGC
*GAPDH*	Forward primer	CAGGAGGCATTGCTGATGAT
	Reverse primer	GAAGGCTGGGGCTCATTT

Abbreviations: miR, microRNA; RT, reverse transcriptase; MELK, maternal embryonic leucine zipper kinase; GAPDH, glyceraldehyde-3-phosphate dehydrogenase.

### Cell culture and transfection

HeLa cells were purchased from the American Type Culture Collection and cultured in Dulbecco modified Eagle medium (Gibco) supplemented with 10% fetal bovine serum (Sijiqing) and 100 U/mL penicillin/streptomycin at 37 °C in a humidified atmosphere containing 5% carbon dioxide. miR-557 mimic, miR-557 inhibitor, negative control (NC)-mimic, and NC-inhibitor (GenePharma) were transfected into the cells using Lipofectamine 3000 reagent (Invitrogen) according to the manufacturer’s instructions.

### Luciferase reporter assay

Wild-type *MELK* 3′ untranslated region (3′UTR; WT) and mutant *MELK* 3′UTR (Mut) constructs were generated in the pGL4.10 vector (Promega). HeLa cells were co-transfected with miR-557 mimic or NC-mimic and either the WT or Mut reporter construct using Lipofectamine 3000 reagent. At 48 hours after transfection, the cells were lysed, and luciferase activity was measured using a dual-luciferase reporter assay kit (Promega).

### Western blotting

Total proteins were extracted, and protein concentrations were determined using a bicinchoninic acid assay kit (Beyotime). Proteins were separated by 12% sodium dodecyl sulfate-polyacrylamide gel electrophoresis. After blocking with 5% nonfat milk at 37 °C for 1 hour, the membranes were incubated with primary antibodies overnight at 4 °C, followed by horseradish peroxidase-conjugated secondary antibodies for 1 hour at room temperature. The primary antibodies used were anti-MELK antibody (#2274; Cell Signaling Technology) and anti-GAPDH antibody (FI01101; Guangzhou Huijun Biotechnology).

### Statistical analysis

Statistical analyses were performed using SPSS, version 26.0, software (SPSS, Chicago, IL, USA), and graphs were generated using GraphPad Prism, version 9.0 (GraphPad Software, La Jolla, CA, USA). The Student *t* test was used to compare continuous variables between 2 groups, and the ꭓ^2^ test was used to compare categorical variables. Pearson correlation analysis was performed to assess the correlations of serum miR-557 and MELK mRNA levels with SCCA and CA125 levels in patients with cervical cancer. Receiver operating characteristic (ROC) curve analysis was used to evaluate the diagnostic value of miR-557 and MELK for cervical cancer. A 2-sided *P* < .05 was considered statistically significant.

## Results

### Clinical features

The clinical characteristics of the 35 patients with cervical cancer and 35 women undergoing routine outpatient physical examinations are presented in Table 2. At initial diagnosis and before any therapeutic intervention, patients with cervical cancer had substantially higher serum SCCA and CA125 levels than did the control group. However, no statistically significant between-group differences were observed in age, number of deliveries, prevalence of hypertension or diabetes, body mass index, age at sexual initiation, white blood cell count, or hemoglobin level.

**Table 2:** The clinicopathologic factors of control and patients with cervical cancer.

Characteristic	Cervical cancer (*n* = 35)	Control (*n* = 35)	t/χ^2^	*P* value
Age, mean (SD), y	57.0 (9.9)	56.3 (10.2)	0.291	.77
Delivery time, mean (SD)	2.4 (0.6)	2.6 (0.5)	1.515	.13
Hypertension, No. (%)			0.324	.57^a^
Yes	9 (25.7)	7 (20.0)		
No	26 (74.3)	28 (80.0)		
Type 2 diabetes			0.085	.77^a^
Yes	7 (20.0)	8 (22.9)		
No	28 (80.0)	27 (77.1)		
Body mass index, mean (SD)	28.5 (3.6)	27.6 (4.2)	0.963	.34
Age of sexual initiation, No. (%)			0.128	.72^a^
≥18 y	30 (85.7)	31 (88.6)		
<18 y	5 (14.3)	4 (11.4)		
White blood cell count, mean (SD), ×10^9^/L	5.3 (2.4)	5.5 (2.2)	0.363	.72
Hemoglobin, mean (SD), g/L	112.5 (15.2)	114.2 (13.8)	0.490	.63
SCCA, mean (SD), ng/mL	8.8 (5.3)	0.8 (0.4)	8.905	<.001
CA125, mean (SD), U/mL	45.7 (18.5)	14.3 (5.4)	9.639	<.001

Abbreviations: SCCA, squamous cell carcinoma antigen; CA125, cancer antigen 125.

a χ^2^ test.

### Expression of *miR-557* and *MELK* mRNA in the peripheral serum of the 2 groups

Serum *miR-557* expression was much lower in the cervical cancer group than in the control group, whereas *MELK* mRNA expression was much higher in the cervical cancer group. These differences were statistically significant (*P* < .01) (Figure 1).

**Figure 1:**
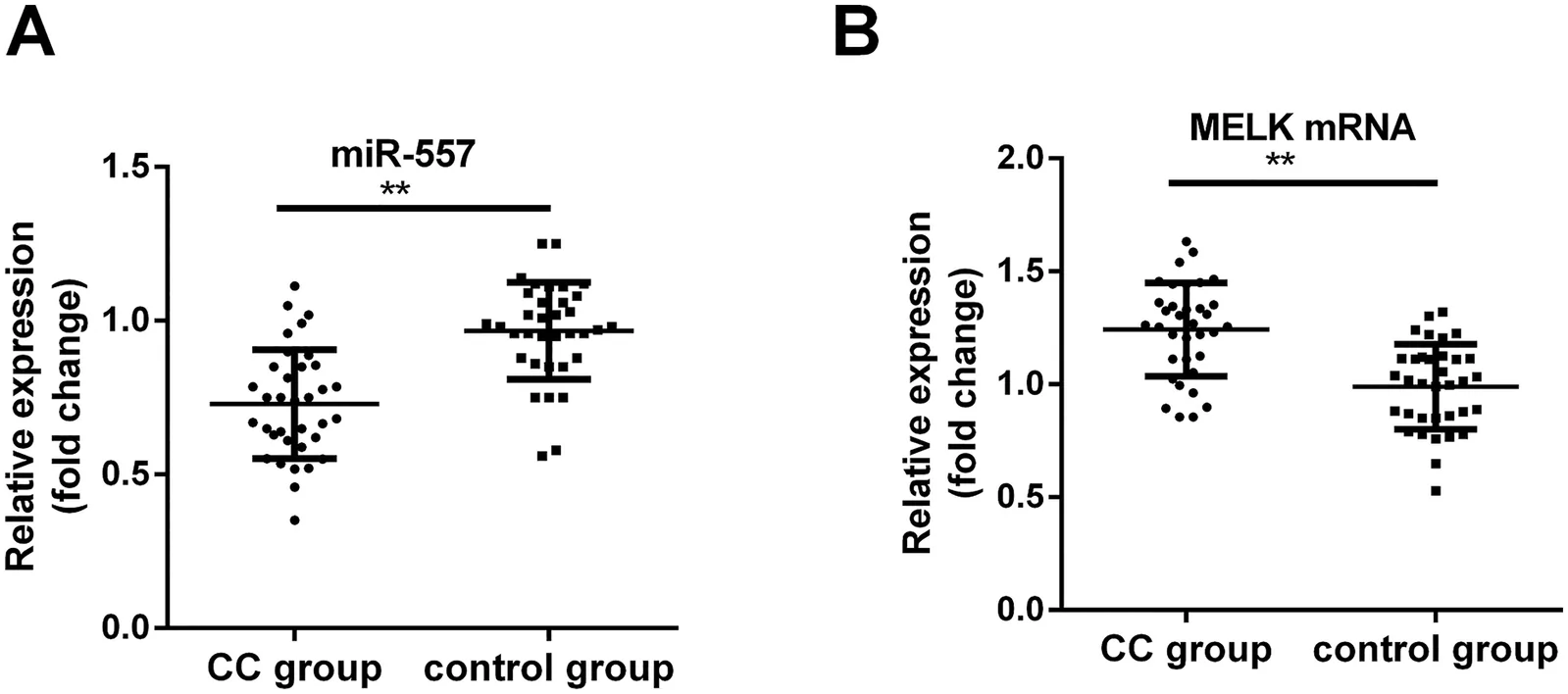
The expression of *miR-557* and *MELK* mRNA in the peripheral serum of the cervical cancer and control groups. **(A)** The expression of *miR-557* in the groups was detected by quantitative RT-PCR. **(B)** The expression of *MELK* mRNA in the 2 groups was detected by quantitative RT-PCR. CC indicates cervical cancer; MELK, maternal embryonic leucine zipper kinase; mRNA, messenger RNA; miR, microRNA; RT-PCR, reverse transcriptase–polymerase chain reaction. **, compared with the control group, *P* < .05.

### Pearson correlation analysis of *miR-557* and *MELK* mRNA expression in patients with cervical cancer

We analyzed the correlations of *miR-557* and *MELK* mRNA expression with SCCA and CA125 levels. Pearson correlation analysis showed that miR-557 expression was weakly and negatively correlated with SCCA (*r* *=* −0.377, *P* = .03) and CA125 (*r* *=* −0.346, *P* = .02). In contrast, *MELK* mRNA expression was weakly and positively correlated with SCCA (*r* *=* 0.467, *P* = .005) and CA125 (*r* *=* 0.520, *P* = .001). Although the correlation coefficients were modest, all associations were statistically significant (Figure 2).

**Figure 2:**
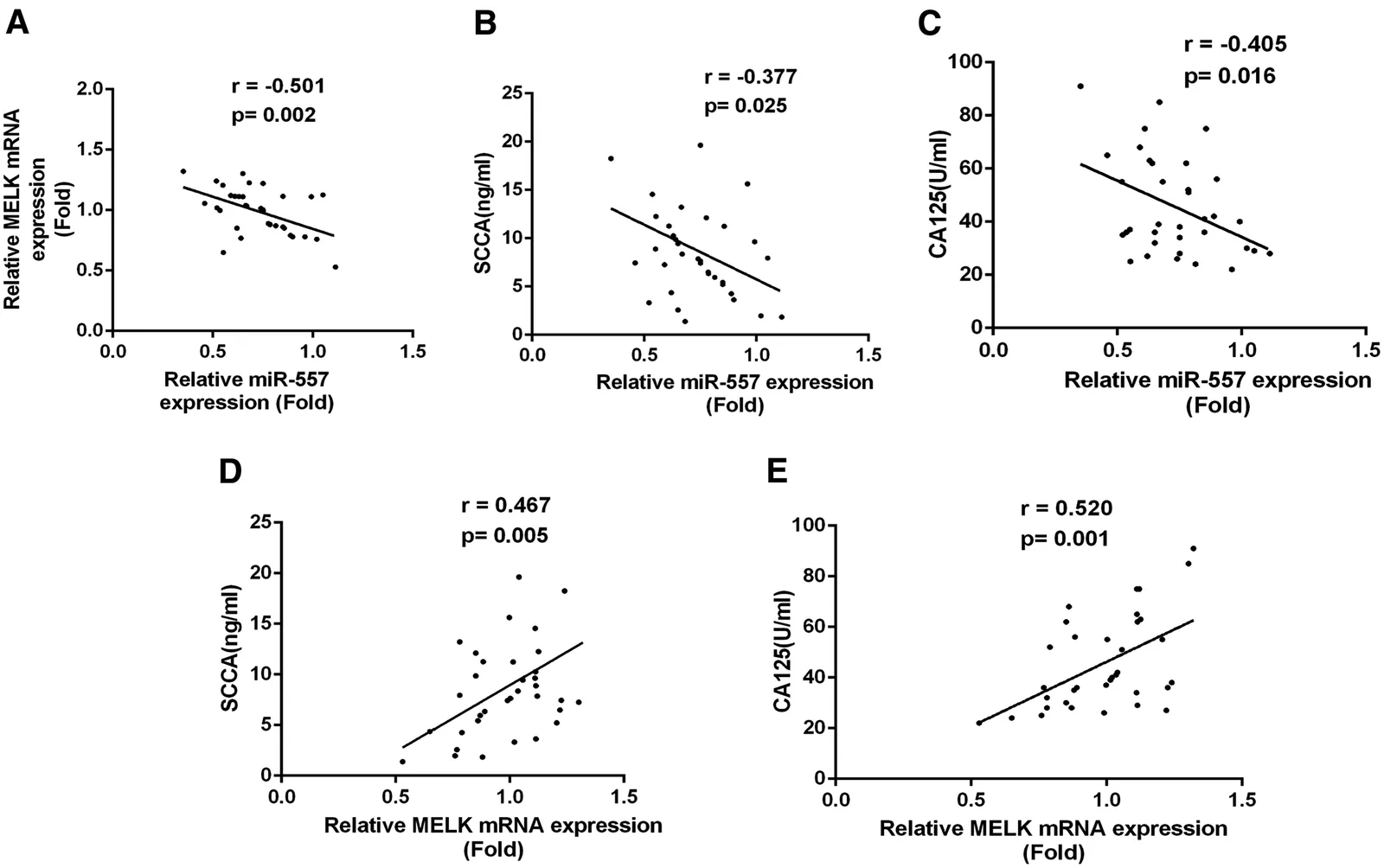
The correlation analysis of serum *miR-557* and *MELK* mRNA with SCCA and CA125 in patients with cervical cancer. **(A)** The expression of *miR-557* in serum is negatively correlated with *MELK* mRNA. **(B)** The expression of *miR-557* in serum is negatively correlated with SCCA. **(C)** The expression of miR-557 in serum is negatively correlated with CA125. **(D)** The expression of *MELK* mRNA in serum is positively correlated with SCCA. **(E)** The expression of *MELK* mRNA in serum is positively correlated with CA125. CA125 indicates cancer antigen 125; *MELK*, maternal embryonic leucine zipper kinase; mRNA, messenger RNA; miR, microRNA; SCCA, squamous cell carcinoma antigen.

### Diagnostic value of *miR-557* and *MELK* in cervical cancer

We performed a ROC curve analysis to evaluate the diagnostic performance of *miR-557* and *MELK* for cervical cancer. The areas under the curve (AUCs) for *miR-557* and *MELK* were 0.836 (80.00% sensitivity and 80.00% specificity) and 0.819 (80.00% sensitivity and 71.40% specificity), respectively. The combined detection of *miR-557* and *MELK* yielded an AUC of 0.909, with a sensitivity of 80.00% and a specificity of 91.40% (Table 3; Figure 3).

**Figure 3:**
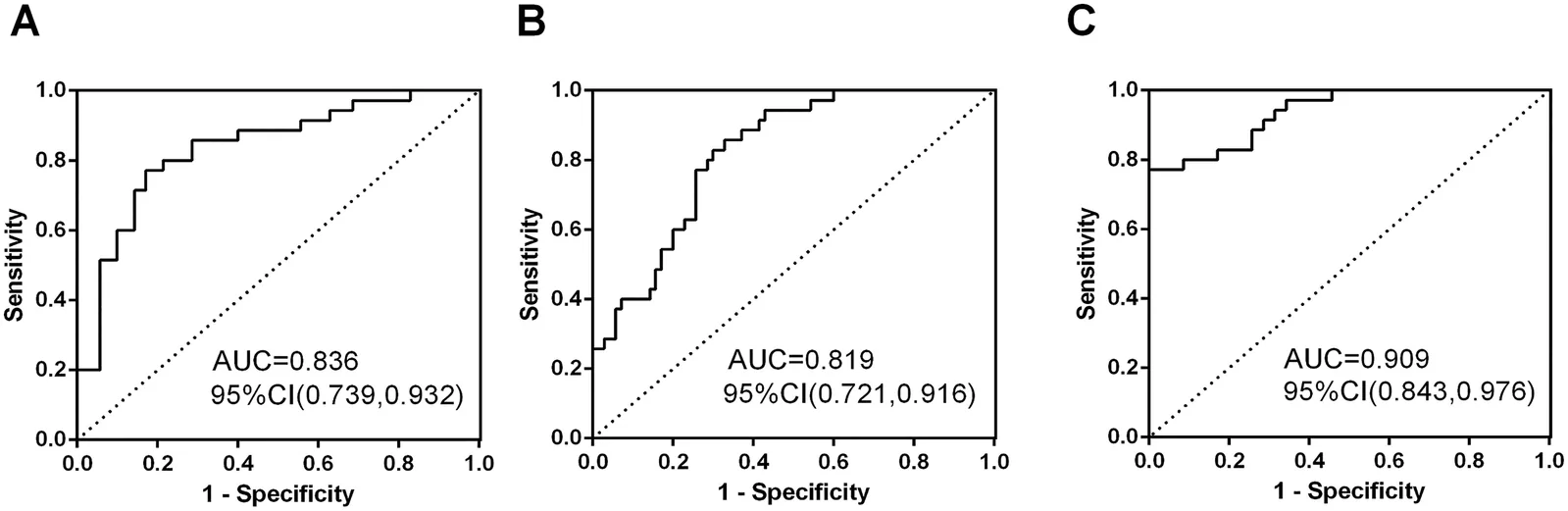
ROC curve for *miR-557* and *MELK*. **(A)** ROC curve for *miR-557*. **(B)** ROC curve for *MELK*. **(C)** ROC curve for the combined use of *miR-557* and *MELK*. AUC indicates area under the curve; *MELK*, maternal embryonic leucine zipper kinase; miR, microRNA; ROC, receiver operating characteristic.

**Table 3:** The diagnostic value of *miR-557* and *MELK* for cervical cancer.

Biomarker	Cutoff value	Sensitivity, %	Specificity, %	Youden index	AUC	95% CI
*miR-557*	0.858	80.00	80.00	60.00	0.836	0.739-0.932
*MELK*	1.123	80.00	71.40	51.40	0.819	0.721-0.916
*miR-557* + *MELK*	—	80.00	91.40	71.40	0.909	0.843-0.976

Abbreviations: miR, microRNA; MELK, maternal embryonic leucine zipper kinase; AUC, area under a curve.

### *MELK* is a direct target of *miR-557*

Based on predictions from the TargetScan database (Whitehead Institute for Biomedical Research) (Figure 4A), we hypothesized that miR-557 directly binds to the 3′UTR of *MELK*. To test this hypothesis, a dual-luciferase reporter assay was performed. Co-transfection of the miR-557 mimic with the *MELK*-3′UTR-WT plasmid substantially reduced luciferase activity, whereas no significant change was observed with the *MELK*-3′UTR-MUT plasmid (Figure 4B). Consistently, transfection with the miR-557 mimic decreased MELK protein expression, whereas transfection with the miR-557 inhibitor increased MELK protein expression (Figure 4C, D). Collectively, these findings support MELK as a direct target of miR-557.

**Figure 4:**
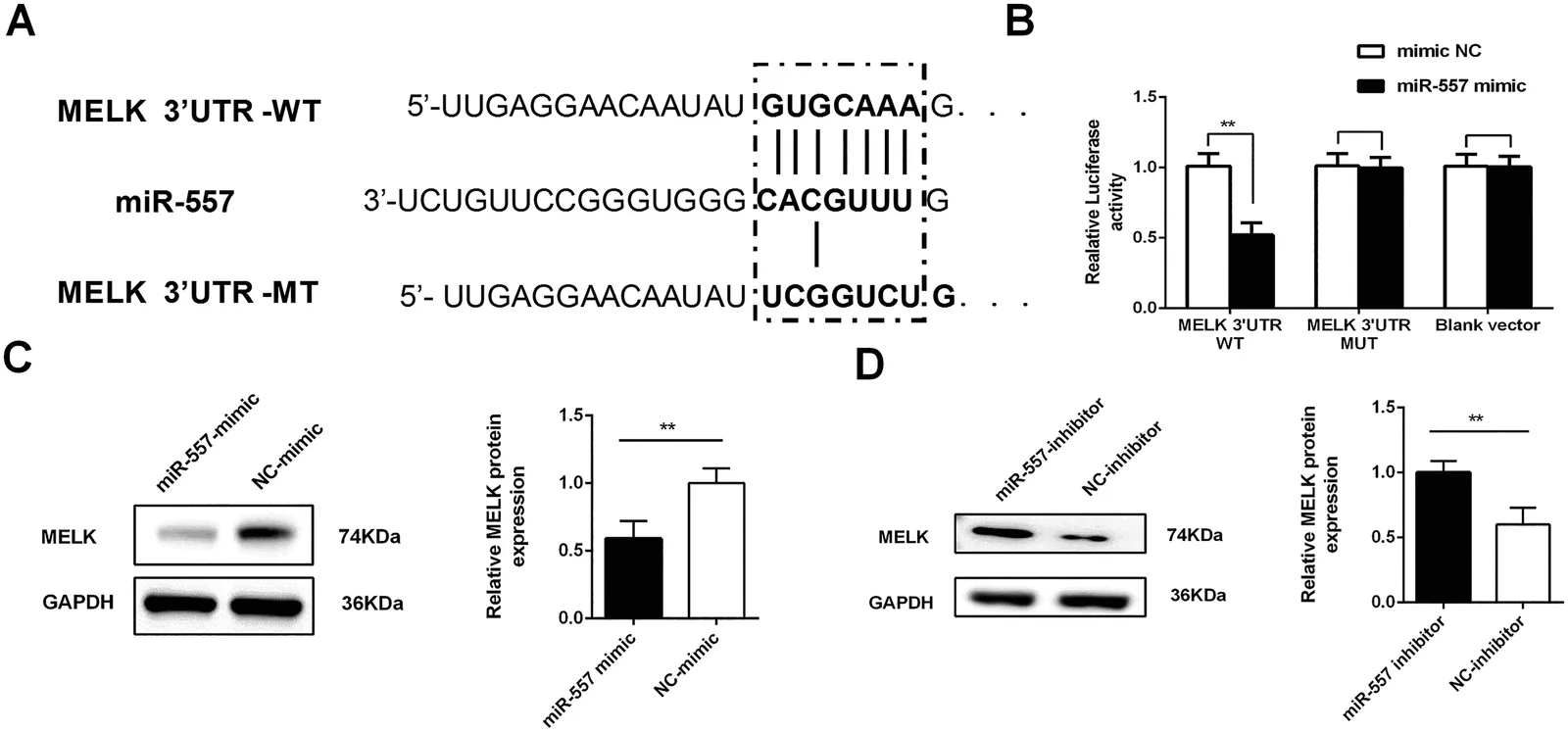
*MELK* is a direct target of *miR-557*. **(A)**  *miR-557* can partially bind with the 3ʹ-UTR of MELK by complementary base pairing, which is predicted using the online bioinformatics tool TargetScan. **(B)** A luciferase reporter gene assay was performed to validate a direct association between *miR-557* and *MELK*. **(C)** After transfection with *miR-557* mimic into HeLa cells, the protein expression levels of *MELK* are measured using Western blotting. **(D)** After transfection with *miR-557* inhibitor into HeLa cells, the protein expression levels of *MELK* are measured using Western blotting. GAPDH indicates glyceraldehyde-3-phosphate dehydrogenase; *MELK*, maternal embryonic leucine zipper kinase; miR, microRNA; MUT, mutant; NC, negative control; WT, wild type. **, compared with the control group, *P* < .05.

## Discussion

Cervical cancer is 1 of the most prevalent malignant tumors among women and is associated with substantial mortality. Screening approaches, including Papanicolaou smears, HPV testing, and co-testing, are routinely used but cannot provide a definitive diagnosis. Biopsy therefore remains the gold standard for histopathologic confirmation.^1^ However, biopsy is invasive and may cause tissue damage. Some patients may tolerate the procedure poorly, and small lesions may be difficult to detect, potentially resulting in missed diagnoses. Current cervical cancer screening approaches also include HPV testing and serum tumor markers such as SCCA and CA125.^8^ However, these methods have limited effectiveness for the early diagnosis of cervical cancer. Previous studies have shown that the combined use of multiple biomarkers can provide high diagnostic sensitivity for cervical cancer.^20^ Consequently, identifying objective and accurate biomarkers for the early diagnosis of cervical cancer remains an important research focus.

MicroRNAs play important roles in regulating gene transcriptase and translation through multiple pathways and thereby influence cancer cell proliferation, apoptosis, invasion, and metastasis.^4^ Increasing evidence is indicating that aberrant miRNA expression contributes to the development of cervical cancer and is associated with tumor metastasis and prognosis, suggesting that miRNAs may represent potential therapeutic targets in this disease. Previous studies have investigated the expression of various miRNAs in patients with cervical cancer. For example, *miR-16*, *miR-25*, *miR-92a*, and *miR-378* were reported to be upregulated, whereas *miR-22*, *miR-27a*, and *miR-100* were downregulated.^21^

Recent studies have shown that miR-557 regulates cell proliferation, differentiation, and apoptosis and is downregulated in various tumor cells, supporting its role as a tumor suppressor.^22^^,^^23^ In osteosarcoma, *miR-557* has been shown to inhibit tumor growth by targeting *KRAS*.^22^ In addition, *miR-557* is expressed at low levels in lung cancer cells and tissues and exerts tumor-suppressive effects by regulating *LEF1* expression.^24^ Feitosa et al^25^ also reported a protective biological role for *miR-557* in cervical cancer. Consistent with these previous findings, our study demonstrated reduced serum *miR-557* expression in patients with cervical cancer. Correlation analysis further revealed weak but statistically significant inverse associations between *miR-557* expression and both SCCA and CA125 levels, suggesting a potential relationship between reduced *miR-557* expression and tumor burden. Serum SCCA and CA125 levels have been associated with disease progression. Ran et al^26^ reported that these markers increased with advancing clinical stage, higher tumor grade, and lymph node metastasis and were substantially elevated in patients with a poor prognosis. Taken together, these findings are consistent with the previously reported tumor-suppressive role of *miR-557* in cervical cancer and extend this evidence to peripheral serum. They further suggest that *miR-557* may be involved in the occurrence and progression of cervical cancer and may have potential as a noninvasive biomarker.

*MELK* is highly expressed in various cancers and has been associated with tumor malignancy.^14‐19^ Previous studies have demonstrated that both *MELK* mRNA and protein levels are upregulated in 4 cervical cancer cell lines, with elevated expression also observed in clinical specimens from patients with cervical cancer.^14^ Notably, *MELK* expression progressively increases from normal cervical tissue through cervical intraepithelial neoplasia grades 1, 2, and 3 to cervical cancer and is associated with disease progression and severity.^27^ In breast and gastric cancers, *MELK* expression has been associated with tumor metastasis and recurrence, suggesting its potential as a prognostic marker and therapeutic target.^28^^,^^29^ In the present study, serum *MELK* levels were much higher in the cervical cancer group than in the control group. Furthermore, *MELK* expression showed weak positive correlations with SCCA and CA125 levels. These findings suggest that increased *MELK* expression may be involved in the development and progression of cervical cancer and may contribute to malignant transformation. Our ROC curve analysis further showed that the AUC for *miR-557* in the diagnosis of cervical cancer was higher than that for *MELK*, whereas the combination of *miR-557* and *MELK* yielded the highest AUC. However, these preliminary findings require validation in larger cohorts. Collectively, these results suggest that *miR-557* and *MELK* have potential diagnostic value for cervical cancer and that their combined assessment may improve diagnostic performance.

The regulatory relationship between miRNAs and mRNAs affects various cancer-related processes, including tumor initiation, progression, drug resistance, and radiosensitivity.^30^ However, the targeted regulatory relationship between *miR-557* and *MELK* has not previously been established. In this study, Pearson correlation analysis of the relative expression levels of *miR-557* and *MELK* mRNA in serum samples revealed a correlation between these 2 molecules. To further investigate this relationship, we used the bioinformatics tool TargetScan, which predicted a potential *miR-557*–binding site within *MELK*. Cellular experiments were subsequently performed to verify the regulatory effect of *miR-557* on *MELK*. Transfection with an *miR-557* mimic altered *MELK* expression, and a dual-luciferase reporter assay further confirmed the direct binding of *miR-557* to *MELK*. These results indicate that *miR-557* may inhibit cervical cancer progression by targeting and regulating *MELK*. This finding further characterizes the molecular relationship between *miR-557* and *MELK* and provides additional insight into the molecular mechanisms underlying cervical cancer.

Our study demonstrates that *miR-557* is expressed at low levels in the serum of patients with cervical cancer, whereas *MELK* expression is elevated. The expression levels of these 2 molecules showed a trend toward a negative correlation and were also associated with serum SCCA and CA125 levels in patients with cervical cancer. Combined detection of *miR-557* and *MELK* improved the diagnostic performance for cervical cancer, suggesting their potential as biomarkers for clinical diagnosis. Cellular experiments further indicated that *miR-557* may inhibit cervical cancer progression by targeting *MELK* expression. These findings characterize the relationship between *miR-557* and *MELK* at the molecular level and provide a basis for further investigation of the pathogenesis of cervical cancer. Despite these findings, however, subgroup analyses according to tumor size or clinical stage were not performed, and HPV subtyping data were unavailable in the present study. Future prospective cohort studies incorporating comprehensive HPV genotyping are therefore warranted to further validate these findings.

## Data Availability

The datasets used and/or analyzed during the current study are available from the corresponding author on reasonable request.
